# A Novel Hybrid Peptide VLP-Aβ Efficiently Regulates Immunity by Stimulating Myeloid Differentiation Protein and Activating the NF-κB Pathway

**DOI:** 10.3390/ijms26125834

**Published:** 2025-06-18

**Authors:** Junyong Wang, Xuelian Zhao, Rijun Zhang, Jing Zhang, Yucui Tong, Zaheer Abbas, Dayong Si, Xubiao Wei

**Affiliations:** Laboratory of Feed Biotechnology, State Key Laboratory of Animal Nutrition and Feeding, College of Animal Science and Technology, China Agricultural University, Beijing 100193, China

**Keywords:** hybrid peptide, immunomodulatory, TLR4/MD2, cyclophosphamide, molecular

## Abstract

Immunosuppression dramatically increases tissue and organ susceptibility to infection, injury, and even cancer. This poses a serious threat to human and animal health. In a previous study, we established a platform for high-throughput design and screening of multifunctional peptides. Using this platform, we successfully identified a novel hybrid peptide, VLP-Aβ (VA), which exhibits both immunomodulatory and antioxidant properties. This study aimed to evaluate the immunomodulatory activity of VA and investigate the underlying molecular mechanisms. In the cyclophosphamide (CTX)-induced immunodeficient mouse model, VA significantly alleviated CTX-induced weight loss. It also restored thymus and spleen indices, and increased serum immunoglobulins (IgA, IgM, IgG) and cytokines (TNF-α, IL-6, IL-1β) levels. VA also improved splenic lymphocyte proliferation, CD4^+^/CD8^+^ T cell ratios, and NK cell cytotoxicity. At the cellular level, western blot analysis showed that VA activated the TLR4-NF-κB pathway in RAW264.7 macrophages. Mechanistically, inhibition of the MD2 protein by L6H21 abolished VA’s immunomodulatory effects. This confirms MD2 as a critical mediator. Molecular docking and dynamics simulations revealed that VA binds stably to the hydrophobic pocket of MD2. These findings suggest that VA exerts immunomodulatory effects by stimulating MD2 and activating the TLR4-NF-κB pathway, which provides new ideas, techniques, and approaches for the development of novel peptide immunomodulators.

## 1. Introduction

The immune system is a critical defense mechanism against infections, inflammation, and autoimmune diseases. It works primarily by recognizing and eliminating foreign pathogens. It consists of two main components: innate immunity and acquired immunity [1,2,3]. The innate immune response plays a crucial role as the first line of defense, recognizing and responding to a wide range of pathogens in a non-specific manner [4,5]. Immunosuppression, which can be temporary or permanent, weakens the immune system. This makes tissues and organs more vulnerable to infections, damage, and even cancer [6,7]. Thus, enhancing immune regulation has been a focus of extensive research.

Peptides have emerged as promising immunomodulators due to their low side effects and diverse functions [6,8,9,10,11,12]. In recent years, immune-regulatory peptides have attracted extensive interest [13,14,15,16,17]. However, the practical application of peptides still faces significant challenges, such as their poor functional diversity, limited biological activity and low extraction or production efficiency. To overcome these challenges, we have established an intelligent platform for the rapid design and screening of peptides with both immunomodulatory and antioxidant properties [18]. Using this platform, we successfully obtained a novel hybrid bifunctional peptide, VLP-Aβ (VA) (Table 1) [18]. However, its detailed immunomodulatory mechanisms remain unclear.

This study aims to evaluate the immunomodulatory activity of VA and explore the underlying molecular mechanisms. The immunomodulatory activity of VA was evaluated using both in vitro models (macrophage RAW 264.7 cells) and in vivo models (cyclophosphamide (CTX)-induced mouse immune deficiency). In the mouse model, VA demonstrated potent immune-regulatory capabilities and mitigated CTX-induced weight loss, reduced immune organ indices, and impaired immune cell function. At the cellular level, VA was also found to activate key proteins in the Toll-like receptor 4–nuclear factor kappa-B (TLR4–NF-κB) signaling pathway, including inhibitory kappa B kinase (IKK), inhibitor NF-κB alpha (IκBα), and NF-κB p65 (p65).

Furthermore, the immunomodulatory effect of VA was abolished when myeloid differentiation protein-2 (MD2) protein was blocked, which demonstrated that the immunomodulatory activity of VA is dependent on MD2. Consistently, molecular docking studies also indicated that VA bind to and interacts with MD2. Taken together, our study provides comprehensive evidence for the immunoregulatory function of VA and elucidates its mechanism of action, providing new insights into the potential applications of immunoregulatory peptides in the food, pharmaceutical and feed industries.

## 2. Results

### 2.1. Hybrid Peptide VA Alleviates Cyclophosphamide-Induced Immunodeficiency in Mice

**Figure 1:**
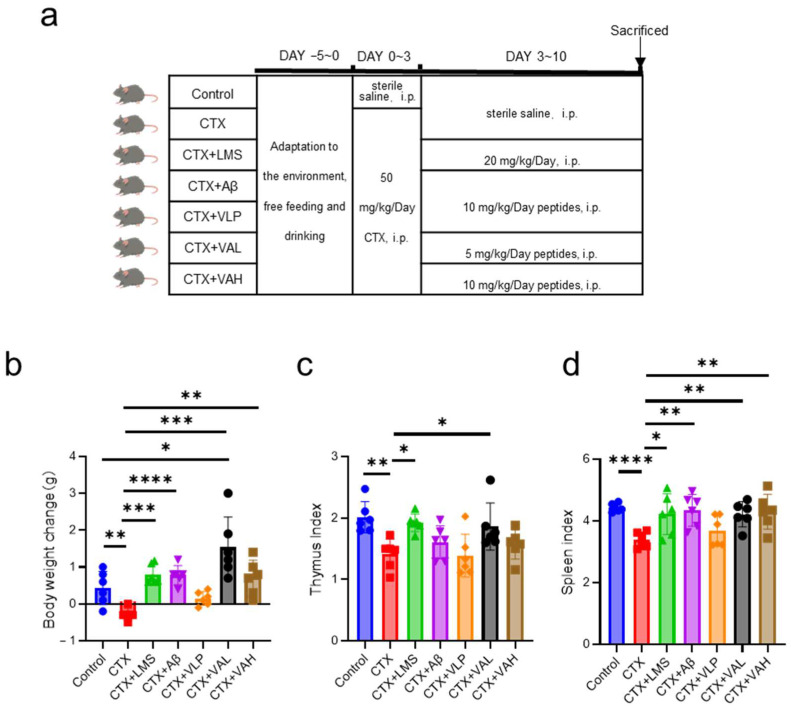
Hybrid peptide VA alleviates CTX-induced immunodeficiency in mice. (**a**) Experimental design for the animal study. Male BALB/c mice (6 weeks old) were divided into 7 groups (*n* = 6 per group). (**b**–**d**) Effects of VA on body weight (**b**), thymus index (**c**), and spleen index (**d**) in cyclophosphamide-treated mice. Data are presented as mean ± SEM (*n* = 6). * *p* ≤ 0.05, ** *p* ≤ 0.01, *** *p* ≤ 0.001, **** *p* ≤ 0.0001.

We investigated the immunomodulatory effects of VA in vivo using a cyclophosphamide (CTX)-induced immunodeficient mouse model (Figure 1a). CTX treatment resulted in decreased body weight and immune organ indices throughout the experimental period (Figure 1b–d). Levamisole (LMS; positive control) and the parent peptide Aβ partially alleviated these effects, although the effect of Aβ was weaker than that of LMS (Figure 1b–d). Both the CTX + VAL group (5 mg/kg/day body weight) (VAL group) and CTX + VAH group (10 mg/kg/day body weight) (VAH group) significantly reversed CTX-induced weight loss (Figure 1b), thymus index (Figure 1c), and spleen index (Figure 1d). Notably, in terms of body weight, VA not only mitigated CTX-induced weight loss, but also promoted weight gain compared to the control group (*p* < 0.05) (Figure 1b).

**Figure 2:**
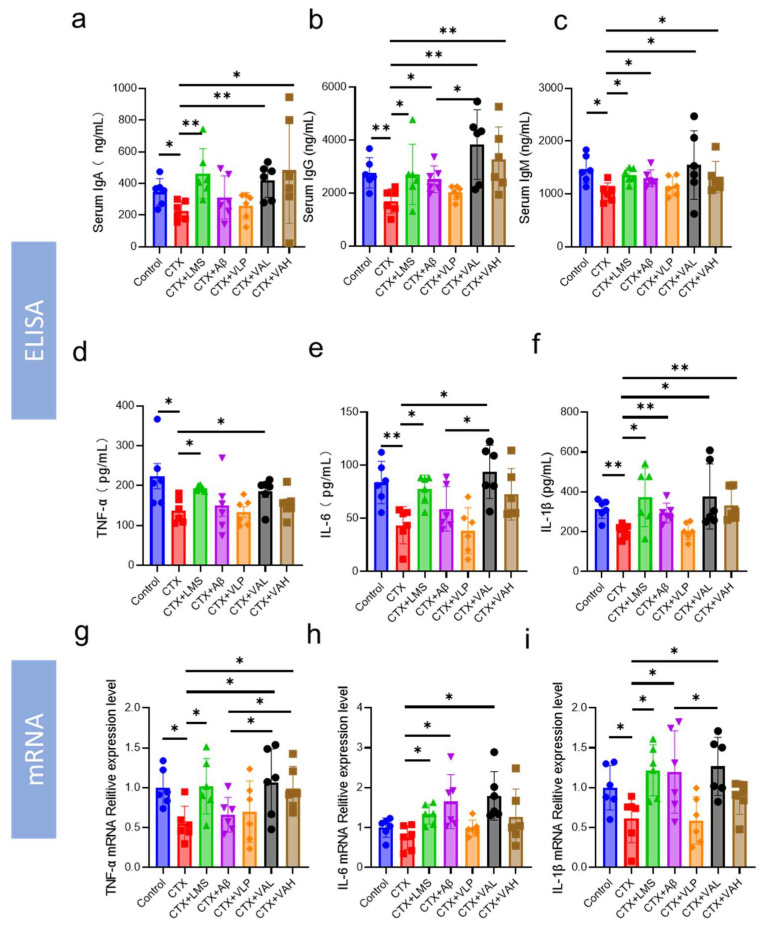
Hybrid peptide VA enhances cytokine and immunoglobulin expression in CTX-induced immunodeficiency mice. (**a**–**f**) Effect of VA on serum IgA (**a**), IgG (**b**), IgM (**c**), TNF-α (**d**), IL-6 (**e**), and IL-1β (**f**) levels. (**g**–**i**) Effect of VA on spleen TNF-α (**g**), IL-6 (**h**), and IL-1β (**i**) mRNA expression. Data are presented as mean ± SEM (*n* = 6). * *p* ≤ 0.05, ** *p* ≤ 0.01.

We also assessed the levels of immunoglobulins (IgA, IgM, IgG) and immunoregulatory factors (TNF-α, IL-6, IL-1β) in the serum of the mice (Figure 2a–f). The results showed that both the VAL and VAH treatments significantly increased IgA, IgG, IgM, and IL-1β levels (*p* < 0.05). As for TNF-α and IL-6, only the VAL group showed significantly higher levels, while the VAH group showed an increasing trend (Figure 2d,e). Notably, the VAL group exhibited a significantly stronger immunomodulatory effect than the Aβ group on IgG and IL-6 expression (Figure 2b,e). Further analysis of mRNA expression levels of TNF-α, IL-6, and IL-1β in spleen tissues (Figure 2g–i) showed similar trends to ELISA results (Figure 2d–f). VAL significantly increased mRNA expression of all three cytokines (Figure 2g–i), and VAH significantly elevated TNF-α expression (Figure 2g).

**Figure 3:**
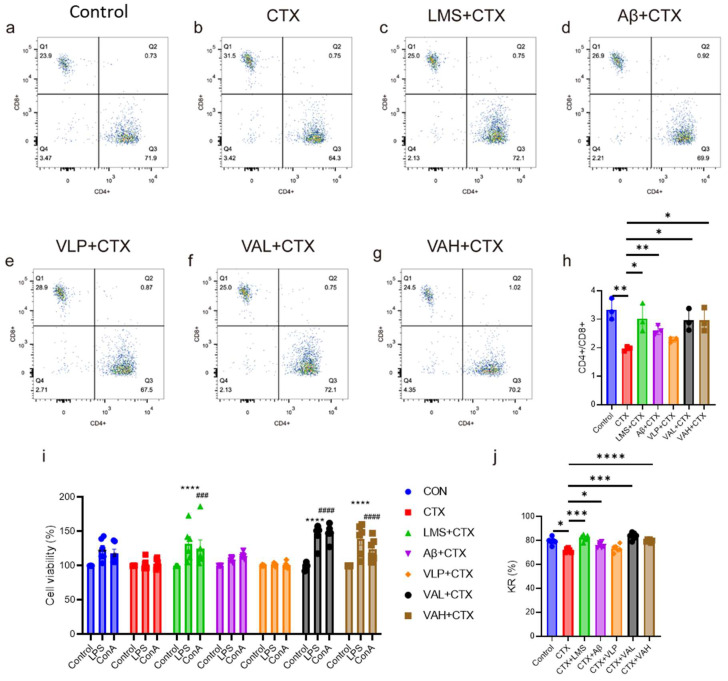
Hybrid peptide VA restores splenic immune cell activity in CTX-induced immunodeficiency mice. (**a**–**g**) Flow cytometric analysis of different splenic T-cell subsets. (**h**) Quantification of the CD4⁺/CD8⁺ ratio. (**i**) Effect of VA on splenocyte proliferation, ** *p* < 0.01, **** *p* < 0.0001, vs. CTX group stimulated with LPS; ### *p* < 0.001 #### *p* < 0.0001, vs. CTX group stimulated with ConA. (**j**) Effect of VA on splenic NK cell cytotoxicity. Data are presented as mean ± SEM (*n* > 3). * *p* ≤ 0.05, ** *p* ≤ 0.01, *** *p* ≤ 0.001, **** *p* ≤ 0.0001.

We further evaluated the immunoregulatory effects of VA on splenic immune cells (Figure 3). Flow cytometric analysis of splenic T cell subpopulations revealed that CTX treatment significantly reduced the ratio of CD4^+^/CD8^+^ cells (Figure 3b,h). This effect was reversed by the LMS, Aβ, VAL, and VAH treatments (Figure 3c,d,f–h). Additionally, lymphocyte proliferation assays showed that CTX impaired the proliferative response to lipopolysaccharide (LPS) and Concanavalin A (ConA) (Figure 3i). In contrast, the LMS, VAL, and VAH groups restored lymphocyte proliferation, even exceeding that of the control group (Figure 3i). Interestingly, Aβ did not significantly alter lymphocyte proliferation. Finally, we assessed the cytotoxicity of splenic natural killer (NK) cell using K562 cells as target cells. CTX treatment significantly reduced NK cell cytotoxicity, while both the VAL and VAH groups significantly increased NK cell killing rates (Figure 3j).

### 2.2. Hybrid Peptide VA Enhances Immunity by Activating the NF-κB Signaling Pathway

**Figure 4:**
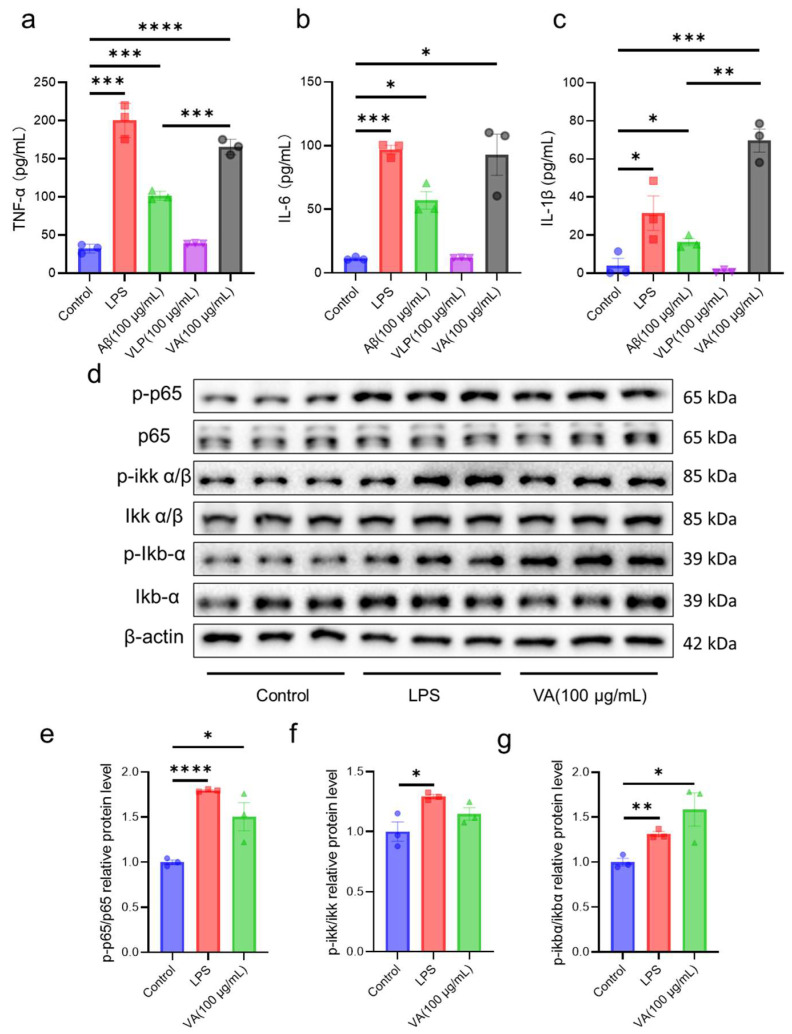
Hybrid peptide VA enhances immunity by activating the NF-κB signaling pathway. (**a**–**c**) Effect of VA on the production of TNF-α (**a**), IL-6 (**b**), and IL-1β (**c**) in RAW264.7 cells. (**d**) Western blot analysis of p65, p-p65, IKKα/β, p-IKKα/β, IκBα, and p-IκBα expression in RAW264.7 cells. (**e**–**g**) Relative quantification of western blot results for p-p65/p65 (**e**), p-IKKα/β/IKKα/β (**f**), and p-IκBα/IκBα (**g**). Data are presented as mean ± SEM (*n* = 3). * *p* ≤ 0.05, ** *p* ≤ 0.01, *** *p* ≤ 0.001, **** *p* ≤ 0.0001.

Through the CTX-induced immunodeficiency model, we demonstrated that VA exhibits dose-dependent immunomodulatory activity. To further explore the potential mechanisms underlying VA’s immune regulation, we assessed the changes in key immunomodulatory factors and downstream signaling proteins in VA-treated RAW264.7 cells (Figure 4). Specifically, we analyzed the expression levels of TNF-α, IL-6, and IL-1β (Figure 4a–c). The results showed that VLP had no significant effect on any of these factors or their phosphorylation levels. In contrast, Aβ and VA significantly increased the expression of TNF-α and IL-1β compared to the control group (Figure 4a–c), which is consistent with our mouse model results (Figure 2d–i). Notably, VA induced a significantly higher level of TNF-α and IL-1β activation compared to its parent peptide Aβ (Figure 4a,c).

We further investigated the effect of VA on the NF-κB signaling pathway by examining the phosphorylation levels of p65, IKKα/β, and IκB-α in RAW 264.7 cells (Figure 4d). The quantification of these proteins is summarized in Figure 4e–g. The results revealed that VA significantly increased the phosphorylation of p65 and IκB-α (Figure 4d,e,g). Although there was no statistically significant difference in IKKα/β phosphorylation between VA and the control group (Figure 4f), immunoblotting results suggested that VA could still enhance IKKα/β phosphorylation (Figure 4d).

### 2.3. Hybrid Peptide VA Activates the NF-κB Pathway in an MD2-Dependent Manner

**Figure 5:**
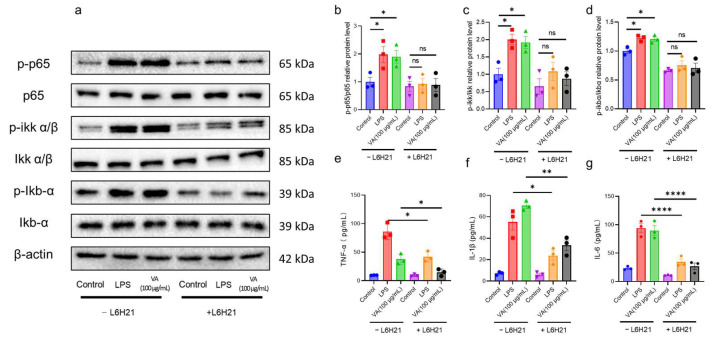
Hybrid peptide VA activates the NF-κB pathway in an MD2-dependent manner. (**a**) Western blot analysis of p65, p-p65, IKKα/β, p-IKKα/β, IκBα, and p-IκBα expression in RAW264.7 cells pretreated with the MD2 inhibitor L6H21. (**b**–**d**) Relative quantification of western blot results for p-p65/p65 (**b**), p-IKKα/β/IKKα/β (**c**), and p-IκBα/IκBα (**d**). (**e**–**g**) Effect of VA on the production of TNF-α (**e**), IL-6 (**f**), and IL-1β (**g**) in RAW264.7 cells pretreated with L6H21. −L6H21 represents the treatment group without L6H21 addition, while +L6H21 denotes the treatment group with L6H21 addition. Data are presented as mean ± SEM (*n* = 3). * *p* ≤ 0.05, ** *p* ≤ 0.01, **** *p* ≤ 0.0001.

Our in vitro and in vivo studies indicate that VA exerts immunomodulatory effects through the NF-κB pathway. However, the exact mechanism of how VA activates the NF-κB pathway remains unclear. To address this, we examined the TLR4-NF-κB signaling pathway in RAW 264.7 cells. We found that L6H21, an MD2 inhibitor, effectively blocked the binding of LPS to MD2 and reduced the expression levels of TNF-α, IL-6, and IL-1β induced by LPS (Figure 5a,e–g). Similarly, after L6H21 treatment, VA’s activation of the NF-κB pathway was almost completely abolished (Figure 5a–d), and the expression of immunomodulatory factors TNF-α, IL-6, and IL-1β was also inhibited (Figure 5e–g). These results suggest that VA exerts its immunomodulatory effects by binding to MD2 and activating the NF-κB signaling pathway.

### 2.4. Hybrid Peptide VA Exerts Immunoregulatory Effects by Binding to the MD2 Hydrophobic Pocket

**Figure 6:**
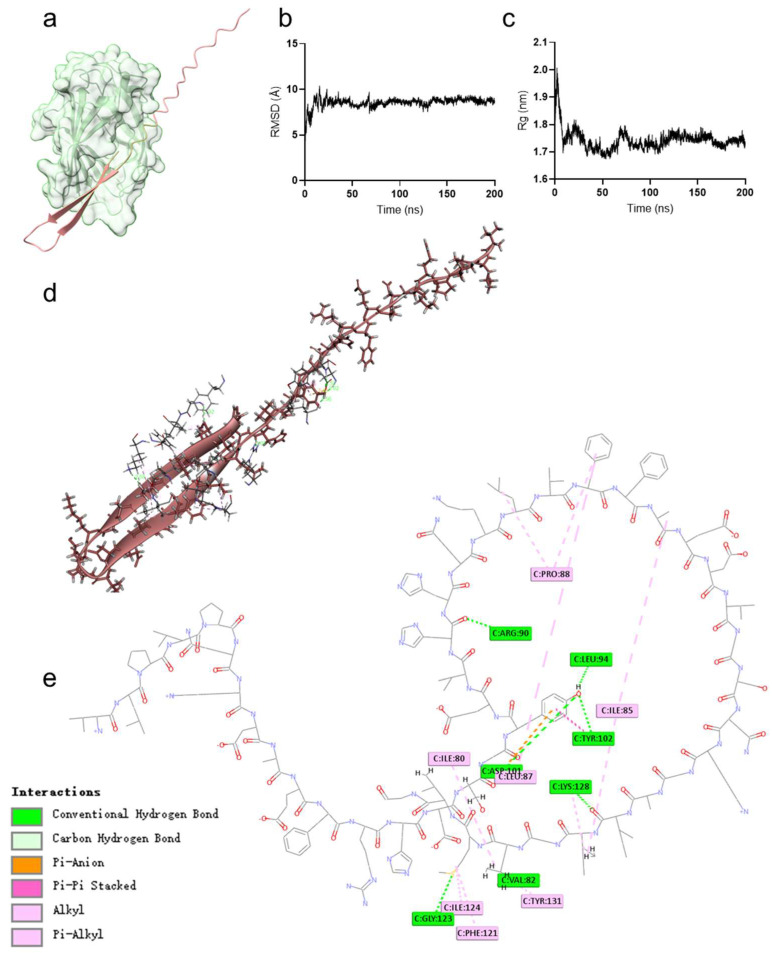
Hybrid peptide VA exerts immunoregulatory effects by binding to the hydrophobic pocket of MD2. (**a**) Overall 3D conformation of VA docked to MD2. (**b**) Root mean square deviation (RMSD) and (**c**) radius of gyration (Rg) from molecular dynamics simulations. (**d**) Local 3D interaction conformation and (**e**) 2D interaction conformation of VA docked to MD2.

We also employed molecular docking and molecular dynamics simulations to analyze the detailed interaction between VA and MD2. The results showed that VA binds to the hydrophobic pocket of MD2 (Figure 6a). Molecular dynamics simulations revealed that the root mean square deviation (RMSD) value of the docked complex remained stable at 8 Å after 200 ns (Figure 6b). The radius of gyration (Rg) remained stable between 1.7 and 1.8 nm during the 200 ns simulation, indicating a stable conformation of the VA-MD2 complex (Figure 6c). Further analysis of the molecular interactions between VA and MD2 revealed multiple interactions, including seven hydrogen bonds, one electrostatic interaction, and ten hydrophobic interactions (Figure 6d,e, Table 2).

## 3. Discussion

Immunosuppression is a temporary or permanent immune dysfunction that increases the susceptibility of tissues and organs to infection, damage, and even cancer, posing a serious threat to human health and the productivity of economic animals [6,7]. Consequently, the development of effective immune modulators has been a hot research topic. In previous work, we designed and screened a hybrid peptide, VA, with both immune-regulatory and antioxidant activities [18]. In this study, we investigate the immune-modulating functions of VA through in vitro and in vivo experiments.

Cyclophosphamide (CTX), an alkylating anticancer agent, is widely used in cancer treatment [19,20]. Due to its bone marrow suppression and immunosuppressive side effects, it is also commonly used to induce immunosuppression in mouse models [21]. CTX treatment damages normal cells, as evidenced by a characteristic reduction in body weight [22,23,24]. As shown in Figure 1b, mice in the CTX group experienced significantly greater weight loss than control the group (*p* < 0.01). Furthermore, the CTX group diminished the weight of immune organs (Figure 1c,d), confirming the successful establishment of the immunosuppressed mouse model. VA, especially at low doses, significantly attenuated CTX-induced decreases in body weight and immune organ indices (Figure 1b–d). This enhanced effects observed in the low-dose VA group suggest a non-linear dose-response, which may involve feedback mechanisms or receptor-level regulation.

CTX also reduces cytokine and immunoglobulin levels [25,26]. Cytokines, such as TNF-α, IL-6, and IL-1β, are critical for maintaining and restoring immune homeostasis, activating immune cells, and triggering further cytokine production [27]. Immunoglobulins (e.g., IgG, IgM, IgA) are essential components of the immune defense, recognizing and neutralizing pathogens, activating the complement system, and promoting phagocytosis [28,29]. Many immune modulators can reverse the decline in cytokines and immunoglobulins caused by CTX [26,30]. In our serum immunological indices analysis, VA similarly increased these factors (Figure 2a–f). In addition, VA upregulated the mRNA expression of TNF-α, IL-6, and IL-1β in the spleen, suggesting that it protects splenic function in this immunosuppression model (Figure 2g–i).

As a central immune organ, the spleen houses a variety of immune cells—including T cells, B cells, NK cells, and dendritic cells, which coordinate with each other to maintain immune homeostasis and response to pathogen invasion [31]. The proliferation of splenic lymphocytes (T and B cells) in response to antigens or mitogens is a fundamental measure of the non-specific immune response [32]. Under normal conditions, both LPS and ConA stimulation lead to increased cellular proliferation [33], but CTX treatment impairs this proliferative capacity. However, we found that VA significantly enhanced lymphocyte proliferation (*p* < 0.001), suggesting that CTX-induced immunosuppression of splenic lymphocytes in mice was alleviated (Figure 3i). T cells are one of the most important immune cells in the immune system, primarily responsible for specific cellular immunity [34]. CD3^+^ T cells represent the overall level of T lymphocytes, among which CD4^+^ and CD8^+^ T cells are the two most critical subgroups [35]. Numerous studies use the CD4^+^/CD8^+^ ratio as an index of overall immune status [36]. An increase in the CD4^+^/CD8^+^ ratio indicates an enhanced immune response, while a decrease in this ratio suggests weakened immune responses. To explore the impact of VA on cellular immunity, we used flow cytometry to assess CD4^+^ and CD8^+^ T cell counts as an indicator of the immune phenotype of splenic cells. As shown in Figure 3a–h, CTX treatment led to a decrease in the CD4^+^/CD8^+^ ratio compared to the control group, which is consistent with previous reports indicating a decline in lymphocyte activity due to CTX treatment [6,36]. Both concentrations of VA significantly increased this ratio, further supporting the effect of VA in enhancing lymphocyte immune activity. NK cells are ideal effector cells in the body, capable of lysing and eliminating tumor cells and virus-infected cells without requiring antigen stimulation or antibodies [37,38]. NK cell cytotoxicity is another important indicator of the body’s immune strength [33]. We assessed the cytotoxicity of splenic NK cells in a CTX-induced immunosuppression model using K562 target cells. The results showed that VA could improve the decreased NK cell cytotoxicity induced by CTX treatment (Figure 3j).

Collectively, VA exhibited strong immunomodulatory activity in immunodeficient mice, alleviating both weight loss and immune organ damage, as well as boosting splenic immune cell functionality. To elucidate the mechanisms behind VA’s immunomodulatory effects, we conducted in-depth cellular studies. VA stimulation of RAW 264.7 cells promoted the secretion of TNF-α, IL-6, and IL-1β (Figure 4a–c), consistent with our in vivo observations (Figure 2d–i). The expression of these cytokines is usually regulated by the NF-κB signaling network, and the Toll-like receptors (TLRs) family is an important component of this network [39]. TLR4, in particular, is the first identified and most extensively characterized TLR. LPS activates TLR4 and triggers downstream signaling through myeloid differentiation primary response protein 88 (MYD88)-dependent and TIR domain-containing adaptor-inducing interferon-β (TRIF)-dependent pathways [40,41]. The MYD88-dependent pathway activates IKK and mitogen-activated protein kinases (MAPK), where the IKK complex (IKKα, IKKβ, and IKKγ) phosphorylates IκB proteins, leading to NF-κB nuclear translocation and subsequent expression of cytokines (TNF-α, IL-6, IL-1β) [41,42]. We found that VA activated key proteins in the MYD88-dependent pathway and upregulated TLR4-NF-κB signaling (Figure 4d–g). However, the precise mechanism behind VA’s activation of this pathway remains unclear.

LPS initiates TLR4 signaling by binding to CD14 and then interacts with the TLR4/MD2 complex, specifically docking within the hydrophobic pocket of MD2, leading to a conformational shift in TLR4 to complete signal transduction [43]. We hypothesized that VA’s immunoregulatory function might similarly involve binding to MD2. Indeed, when we blocked the hydrophobic pocket of MD2 using the MD2 inhibitor L6H21 [44], VA-mediated TLR4-NF-κB activation was almost completely abrogated, and the induction of TNF-α, IL-6, and IL-1β was significantly reduced (Figure 5). These findings indicate that VA’s immune modulation depends on MD2. Numerous studies of MD2-binding molecules (e.g., lipid A analogs, natural products, peptides) have shown that their immunomodulatory or anti-inflammatory functions are the result of interactions with the MD2 hydrophobic pocket [45,46,47]. To further clarify how VA interacts with MD2, we performed molecular dynamics simulations and docking experiments (Figure 6). VA localized to the MD2 hydrophobic pocket. Previously identified key residues for ligand binding include Arg90, Glu92, Tyr102, Ser120, Phe121, Lys122, Ile124, and Lys128 [11,44,46,48,49,50]. In the VA–MD2 interaction, VA forms hydrogen bonds with Arg90, Leu94, Asp101, Tyr102, Gly123, and Lys128 of MD2. Notably, while Arg90, Tyr102, and Lys128 were already known, Leu94, Asp101, and Gly123 had not been reported in MD2-ligand interactions. Combining our in vivo and in vitro findings, we propose that Leu94, Asp101, and Gly123 may play critical roles in the interaction of MD2 with exogenous small molecules and thus deserves further investigation. Although the binding interaction between VA and MD2 was determined through the MD2 inhibitor L6H21 and molecular docking simulations, this study still requires further analysis of the specificity of the binding between VA and MD2.

Taken together, these findings suggest that VA is an extremely promising novel immunomodulator and provide a solid foundation for its further application in the food, pharmaceutical and feed industries. However, several limitations of the present study should be acknowledged. Specifically, the pharmacokinetics, long-term efficacy, and validation in human cell models remain to be investigated in future work. Furthermore, a comprehensive assessment of its biosafety and toxicological profile through systematic in vivo experiments is also warranted.

## 4. Materials and Methods

### 4.1. Chemicals and Reagents

The peptides Aβ, VLP, and VA were synthesized by GL Biochem Ltd. (Shanghai, China) at 95% purity. All peptide stock solutions were fully dissolved in DMSO and diluted to the desired working concentrations as required during experimental procedures. RAW 264.7 and K562 cells were obtained from the Shanghai Cell Bank, Institute of Cell Biology, Chinese Academy of Sciences (Shanghai, China). Dulbecco’s Modified Eagle’s Medium (DMEM) and Iscove’s Modified Dubecco’s Mediumwas (IMDM) were purchased from Gibco (Waltham, MA, USA). Fetal bovine serum (FBS) was obtained from Procell (Wuhan, China). Penicillin-streptomycin, RIPA buffer, lipopolysaccharide (LPS), CCK-8, BCA, TNF-α, IL-6, IL-1β, IgA, IgG, and IgM assay kits were purchased from Beijing Solarbio Science & Technology Co., Ltd. (Beijing, China). Levamisole was purchased from Sigma-Aldrich (St. Louis, MO, USA). ConA and L6H21 were purchased from MedChemExpress (Shanghai, China). ECL reagent was purchased from Beyotime Biotechnology (Shanghai, China). TRIzol reagent, reverse transcription kits, and SYBR mix were purchased from Vazyme (Nanjing, China). All other analytical-grade reagents were obtained from China National Medicines Corporation Co., Ltd. (Beijing, China). FITC-CD4 (cat. no. 11-0041-82), PE-CD3 (cat. no. 12-0031-82), and APC-CD8 (cat. no. 12-0081-82) antibodies were purchased from eBioscience. The NF-κB p65 (cat. no. T55034), phospho-NF-κB p65 (cat. no. TP56372), IKKα/β (cat. no. T55735), phospho-IKKα/β (cat. no. TP56290), IκBα (cat. no. T55026), phospho-IκBα (cat. no. TP56280), and β-actin (cat. no. P30002) antibodies were purchased from Abmart (Shanghai, China).

### 4.2. Cell Culture

RAW 264.7 cells were cultured in DMEM supplemented with 10% FBS and 1% penicillin-streptomycin. The cells were incubated at 37 °C in a humidified atmosphere containing 5% CO_2_ and passaged daily.

K562 cells were cultured in IMDM supplemented with 10% FBS and 1% penicillin-streptomycin. The cells were incubated at 37 °C in a humidified atmosphere containing 5% CO_2_ and passaged every three days.

Spleen lymphocytes were isolated and resuspended in RPMI-1640 medium with 10% FBS and 1% penicillin-streptomycin.

### 4.3. Animal Models

Male BALB/c mice (aged 6 weeks) were purchased from GemPharmatech Co., Ltd. (Nanjing, China) and maintained under specific pathogen-free (SPF) conditions. Mice had free access to food and water. All animal procedures were conducted in accordance with the guidelines of the Institutional Animal Care and Use Committee of China Agricultural University.

Mice were randomly divided into seven groups (six mice per group): the control group, the CTX group (50 mg/kg/day body weight), the CTX + Levamisole (LMS) group (20 mg/kg/day body weight), the CTX + Aβ group (10 mg/kg/day body weight), the CTX + VLP group (10 mg/kg/day body weight), the CTX + VAL group (5 mg/kg/day body weight), and the CTX + VAH group (10 mg/kg/day body weight). After a 5-day acclimatization, mice in all groups except the control were intraperitoneally injected with cyclophosphamide (CTX) at 50 mg/kg/day from Days 1 to 3. The control group received an equivalent volume of saline (0.9%). From Days 4 to 10, mice were treated daily with the specified drugs or peptides (control and CTX groups again received saline). Sixteen hours after the final treatment, mice were sacrificed, and blood, spleen, and thymus tissues were collected. Body weight was recorded at the beginning and at the end of the experiment.

#### 4.3.1. Organ Index Calculation

Fat and connective tissue were carefully removed from the spleen and thymus before weighing. The organ index was calculated as follows:

$$\mathrm{Organ}\;\mathrm{index}\;(\mathrm{mg}/g)=\frac{Organ\;weight}{Body\;weight}$$

#### 4.3.2. ELISA Measurement

Blood samples were centrifuged at 5000 rpm for 20 min at 4 °C to separate the serum. TNF-α, IL-6, IL-1β, IgG, IgM, and IgA levels in the serum were measured using ELISA kits according to the manufacturer’s protocol.

#### 4.3.3. RT-PCR

Spleens were minced, and total RNA was extracted using TRIzol reagent. RNA concentration and purity were determined using a NanoDrop spectrophotometer. First-strand cDNA was synthesized using a commercial reverse transcription kit according to the manufacturer’s protocol. The primers are listed in Table 3. PCR amplification was performed using the following conditions: pre-denaturation at 95 °C for 30 s, denaturation at 95 °C for 5 s, and extension at 60 °C for 30 s for a total of 40 cycles. After amplification, a final denaturation step at 95 °C for 5 s, followed by 60 °C for 60 s and a melting curve at 95 °C for 1 s, was included for a total of 1 cycle. Gene expression was normalized to β-actin, and results were expressed as fold change relative to control samples.

#### 4.3.4. Flow Cytometry Analysis of Spleen T Cell Subpopulations

Spleen lymphocytes were isolated as previously described, and viable cells were counted using trypan blue exclusion [36]. Approximately 1 × 10^6^ cells per sample were incubated with fluorochrome-conjugated antibodies for 30 min at 4 °C in the dark. The antibodies used were FITC-anti-CD4 (cat. no. 11-0041-82), PE-anti-CD3 (cat. no. 12-0031-82), and APC-anti-CD8a (cat. no. 12-0081-82), all purchased from Thermo Fisher Scientific (Waltham, MA, USA), and were used at the manufacturer-recommended dilutions.

After staining, cells were washed twice with PBS and filtered through a 40 μm cell strainer to remove debris and aggregates. Flow cytometry was performed using a BD LSRFortessa™ flow cytometer (Becton, Dickinson and Company, Franklin Lakes, NJ, USA), and data were analyzed with FlowJo software (version 10.0.7). A total of 10,000 events were collected per sample for analysis.

#### 4.3.5. Proliferation Assay for Spleen Lymphocytes

Spleen lymphocytes were isolated, counted by trypan blue staining, and resuspended in RPMI-1640 medium containing 10% FBS and 1% penicillin-streptomycin. A total of 2 × 10^5^ cells/well were seeded in a 96-well plate and incubated overnight. Cells were then treated with LPS (1 μg/mL) or ConA (2.5 μg/mL) for 24 h. Proliferation was assessed using the CCK-8 assay according to the manufacturer’s instructions.

#### 4.3.6. NK Cell Cytotoxicity Assay

Spleen cells served as effector cells, while K562 cells served as target cells. K562 cells (1 × 10^5^ cells/well) were plated in a 96-well plate and incubated overnight. Spleen cells (5 × 10^6^ cells/well) were then added and co-incubated for 4 h. Afterward, CCK-8 reagent was added to each well, and absorbance at 450 nm was measured. NK cell kill rate (KR) was calculated as follows:

$$KR=\left(1-\frac{{OD}_{S}-{OD}_{E}}{{OD}_{T}}\right)\times 100\%$$

where OD_S_ is the absorbance value of the wells with both spleen and K562 cells, OD_E_ is the absorbance value of the wells with only spleen cells, and OD_T_ is the absorbance value of the wells with only K562 cells.

### 4.4. Enzyme-Linked Immunosorbent Assay (ELISA)

RAW 264.7 cells were seeded at 2 × 10^6^ cells per well in a 6-well plate and incubated overnight. The cells were then treated with 100 μg/mL of Aβ, VLP, VA, or 100 ng/mL of LPS for 24 h. The supernatants were collected, and the concentrations of TNF-α, IL-6, and IL-1β were measured using ELISA kits according to the manufacturer’s instructions.

### 4.5. Western Blot Analysis

RAW 264.7 cells were seeded at 2 × 10^6^ cells per well in a 6-well plate and incubated overnight. The cells were then treated with 100 μg/mL of VA or 100 ng/mL of LPS for 6 h. The cells were collected, and proteins were extracted using RIPA buffer, with protease inhibitors added. Protein concentrations were determined using the BCA protein assay kit. Proteins were separated by SDS-PAGE and transferred to a PVDF membrane. Membranes were blocked with 5% non-fat milk, and then incubated overnight with primary antibodies. HRP-conjugated secondary antibodies were used for detection, and the bands were visualized using ECL.

### 4.6. Interaction Analysis of Hybrid Peptide VA with MD2

RAW 264.7 cells were seeded at 2 × 10^6^ cells per well in a 6-well plate and incubated overnight. The cells were co-incubated with 10 μM of the MD2 inhibitor (L6H21), 100 μg/mL of VA, or 100 ng/mL of LPS for 6 h, followed by Western blot (WB) analysis. For ELISA analysis, the co-incubation lasted 24 h, and the cell supernatants were analyzed accordingly.

### 4.7. Molecular Docking and Dynamics Simulation

The 3D structure of the hybrid peptide VA was generated using PEP-FOLD 3.5, and the crystal structure of MD2 was obtained from the Protein Data Bank (PDB ID: 2Z64). Molecular docking and dynamics simulations were performed according to our previous methods [11]. The docking complexes were visualized and analyzed for molecular interactions using Discovery Studio 2021.

### 4.8. Statistical Analysis

Statistical analysis was performed using GraphPad Prism v9.0. All data passed normality verification by the Shapiro-Wilk test, and homogeneity of variance was confirmed by the Brown-Forsythe test. Intergroup differences were analyzed using one-way ANOVA, and the Bonferroni correction was further applied to adjust the significance level for multiple comparisons. All data are presented as mean ± SEM of at least three independent experiments. Statistical significance was considered at *p* ≤ 0.05. NS: *p* > 0.05, *: *p* ≤ 0.05, **: *p* ≤ 0.01, ***: *p* ≤ 0.001, ****: *p* ≤ 0.0001.

## Figures and Tables

**Table 1:** Sequence Information of Peptides.

Peptide	Sequence
Aβ	DAEFRHDSGYEVHHQKLVFFAEDVGSNKGAIIGLMVG
VLP	VLPVPQK
VLP-Aβ	VLPVPQKDAEFRHDSGYEVHHQKLVFFAEDVGSNKGAIIGLMVG

**Table 2:** Ramachandran Plot Analysis of the Conformational Plausibility of Docking Complexes.

Name	Distance	Category	From	To
C:ARG90:HH21–A:HIS20:O	3.07	Hydrogen Bond	C:ARG90:HH21	A:HIS20:O
C:ASP101:HN–A:TYR17:OH	2.02	Hydrogen Bond	C:ASP101:HN	A:TYR17:OH
C:TYR102:HN–A:TYR17:OH	2.57	Hydrogen Bond	C:TYR102:HN	A:TYR17:OH
C:GLY123:HN–A:MET42:SD	2.92	Hydrogen Bond	C:GLY123:HN	A:MET42:SD
C:LYS128:HZ1–A:ILE38:O	2.58	Hydrogen Bond	C:LYS128:HZ1	A:ILE38:O
A:TYR17:HH–C:LEU94:O	1.56	Hydrogen Bond	A:TYR17:HH	C:LEU94:O
C:LYS128:HE2–A:ILE38:O	1.71	Hydrogen Bond	C:LYS128:HE2	A:ILE38:O
C:ASP101:OD2–A:TYR17	3.46	Electrostatic	C:ASP101:OD2	A:TYR17
C:TYR102–A:TYR17	3.79	Hydrophobic	C:TYR102	A:TYR17
C:ILE80–A:LEU41	4.71	Hydrophobic	C:ILE80	A:LEU41
C:ILE85–A:ILE39	5.00	Hydrophobic	C:ILE85	A:ILE39
C:PRO88–A:LEU24	5.43	Hydrophobic	C:PRO88	A:LEU24
C:ILE124–A:MET42	4.27	Hydrophobic	C:ILE124	A:MET42
C:LYS128–A:ILE39	5.12	Hydrophobic	C:LYS128	A:ILE39
A:ALA28–C:ILE85	4.28	Hydrophobic	A:ALA28	C:ILE85
C:PHE121–A:MET42	4.60	Hydrophobic	C:PHE121	A:MET42
C:TYR131–A:LEU41	5.09	Hydrophobic	C:TYR131	A:LEU41
A:PHE26–C:LEU87	4.08	Hydrophobic	A:PHE26	C:LEU87
A:PHE26–C:PRO88	5.00	Hydrophobic	A:PHE26	C:PRO88

**Table 3:** Sequences of the primers used for RT-PCR assays.

Gene		Sequence (5′–3′)	Length
*TNF-α*	F	GGCCAACGGCATGGATCTCAAA	22
	R	TAGCAAATCGGCTGACGGTGTG	22
*IL-6*	F	TCTTGGGACTGATGCTGGTGA	21
	R	TTGGGAGTGGTATCCTCTGTGAA	23
*IL-1β*	F	AATCTCGCAGCAGCACATCAACA	23
	R	ACACCAGCAGGTTATCATCATCATCC	26
*β-actin*	F	TCACTATTGGCAACGAGCGGTTC	23
	R	CAGCACTGTGTTGGCATAGAGGTC	24

## Data Availability

Data are contained within the article.
